# Abnormalities of plasma cytokines and spleen in senile APP/PS1/Tau transgenic mouse model

**DOI:** 10.1038/srep15703

**Published:** 2015-10-27

**Authors:** Seung-Hoon Yang, Jiyoon Kim, Michael Jisoo Lee, YoungSoo Kim

**Affiliations:** 1 Center for Neuro-Medicine, Brain Science Institute, Korea Institute of Science and Technology, Hwarangno 14-gil 5, Seongbuk-gu, Seoul, Republic of Korea; 2 Biological Chemistry Program, Korea University of Science and Technology, 217 Gajungro, Yuseong-gu, Daejeon, Republic of Korea; 3 Department of Medical Education, California Northstate University College of Medicine, 9700 W Taron Drive, Elk Grove, CA 95757, USA

## Abstract

The blood-based diagnosis has a potential to provide an alternative approach for easy diagnosis of Alzheimer’s disease (AD) with less invasiveness and low-cost. However, present blood-based AD diagnosis mainly focuses on measuring the plasma Aβ level because no other biomarkers are found to possess evident transport mechanisms to pass the blood-brain barrier. In order to avoid diagnosing non-demented individuals with Aβ abnormality, finding additional biomarkers to supplement plasma Aβ is essential. In this study, we introduce potential neurodegenerative biomarkers for blood-based diagnosis. We observed severe splenomegaly and structural destruction in the spleen with significantly decreased B lymphocytes in senile APP_swe_, PS1_M146V_ and Tau_P301L_ transgenic mice. We also found that inflammatory cytokines associated with splenic dysfunction were altered in the plasma of these mice. These findings suggest potential involvement of the splenic dysfunction in AD and the importance of biomarker level alterations in the plasma as putative diagnostic targets for AD.

Alzheimer’s disease (AD) is the representative senile disorder characterized by progressive decline of memory and psychiatric manifestations^1^,^2^,^3^,^4^. The obligate neuropathological hallmarks that occur in the AD brain are the deposition of amyloid-β (Aβ) and hyperphosphorylated tau (p-tau)^5^,^6^,^7^,^8^. Neuroimaging techniques such as amyloid position emission tomography (PET) have been assisting diagnosis of AD but numerous cases of non-demented individuals with amyloid-PET positive have complicated the situation^9^,^10^,^11^,^12^,^13^,^14^,^15^. Tau-PET, thus, has been studied as a tool to supplement the amyloid-PET^16^,^17^. However, PET imaging is not only costly and time-consuming but also rarely available to general patients yet^18^,^19^. Blood-based diagnosis targeting AD hallmarks can be a less-invasive and convenient alternative^20^. Lower plasma Aβ(1–42) and increased risk of AD development were found to be associated, which further supports the potential of AD blood-based diagnosis^21^. As seen in non-demented individuals with amyloid-PET positive, Aβ(1–42) will require additional biomarkers at the blood-based level for reliable diagnosis. Unfortunately, plasma tau is difficult to detect since it is unclear whether the blood-brain barrier has any known transport mechanisms for tau proteins yet. Aforementioned limitations suggest the need for development of additional biomarkers, which can assume tau’s role in AD diagnosis at the blood-based level. Recent studies attempted to search for such biomarkers and some have proposed phospholipids and inflammatory proteins in the plasma as useful biomarkers for AD diagnosis^21^,^22^,^23^,^24^.

Triple-transgenic mouse (3xTg-AD) model that encodes independent human APP_swe_, PS1_M146V_ and Tau_P301L_ transgenes exhibits formation of both Aβ plaques and neurofibrillary tau tangles associated with synaptic dysfunction typically observed in AD patients^25^,^26^,^27^. This model also exhibits decreasing Aβ concentration in the CSF in an age-dependent manner as seen in AD patients in clinical trials, offering an opportunity to identify additional blood-based biomarkers for AD. It is notable that the 3xTg-AD mice can have severe splenomegaly and autoantibody generation along with behavioral deficits at 12 months of age^28^. Since splenomegaly often appears with inflammatory signs^29^,^30^,^31^, we hypothesized possible alteration in plasma cytokines due to mutated APP_swe_, PS1_M146V_ and Tau_P301L_ genes.

Here, we searched for plasma biomarker candidates from selected cytokines in the senile 3xTg-AD mice to supplement plasma Aβ(1–42) measurement in both diagnosis and prognosis of AD. The level of Aβ(1–42) was measured in the CSF and plasma of 14- and 24-month-old 3xTg-AD mice to confirm whether the mice can mimic human AD pathophysiology and if the plasma can present the same age-dependent alteration as CSF does. After observing splenomegaly and splenic destruction, we quantitatively measured the levels of each cytokine in the plasma using the Luminex Bio-plex cytokine assay.

## Results

### The level of Aβ(1–42) decreased in the CSF and plasma of 3xTg-AD mice with age

**Figure 1:**
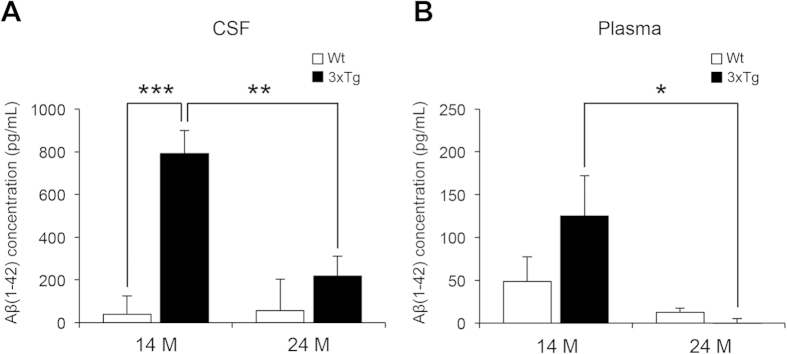
Concentration of Aβ(1–42) in the CSF and plasma from wild-type (Wt) and 3xTg-AD mice at the age of 14 and 24 months. Concentrations of Aβ(1-42) in the CSF (**A**) and plasma (**B**) of 3xTg-AD mice were compared with wild-type (Wt) mice at that age of 14 and 24 months. One-way ANOVA followed by Bonferroni’s post-hoc comparisons tests were performed in all statistical analyses (*P < 0.05, **P < 0.01, ***P < 0.001).

Based on clinical investigations, the level of CSF Aβ(1–42) decreases with age in AD patients, displaying strong correlation with both the clinical diagnosis of AD and amyloid neuropathology in post-mortem brains^32^,^33^,^34^. The 3xTg-AD mice begin to express AD-like neuropathologies and behaviors within 12 months after birth and, therefore, we selected 14- and 24-month-old mice. The concentrations of Aβ(1–42) in the CSF of 14- and 24-month-old senile 3xTg-AD transgenic mice were measured to confirm the transgenic mice’s ability to imitate the human AD pathophysiology. A dramatic decrease in CSF Aβ(1–42) levels in an age-dependent manner was observed in the 3xTg-AD mice. In the 14-month-old groups, the 3xTg-AD mice showed significantly higher Aβ(1–42) level compared with the wild-type, while no statistically significant differences in Aβ(1–42) level were observed between the 24-month-old groups (Fig. 1A). Because of previous cohort studies reporting correlation of lower plasma Aβ(1–42) levels and AD progression^21^, the concentration of Aβ(1–42) in the plasma of 3xTg-AD mice was measured. Similar to the case in CSF, 24-month-old 3xTg-AD mice displayed significantly decreased Aβ(1–42) level in the plasma compared with the 14-month-old mice (Fig. 1B). However, wild-type mice did not display any statistically significant change in Aβ(1–42) level in an age-dependent manner. These results indicate that the 3xTg-AD mice are capable of mimicking the AD pathophysiology in human patients.

### Spleen was extensively enlarged and the structure was destroyed in senile 3xTg-AD mice

**Figure 2:**
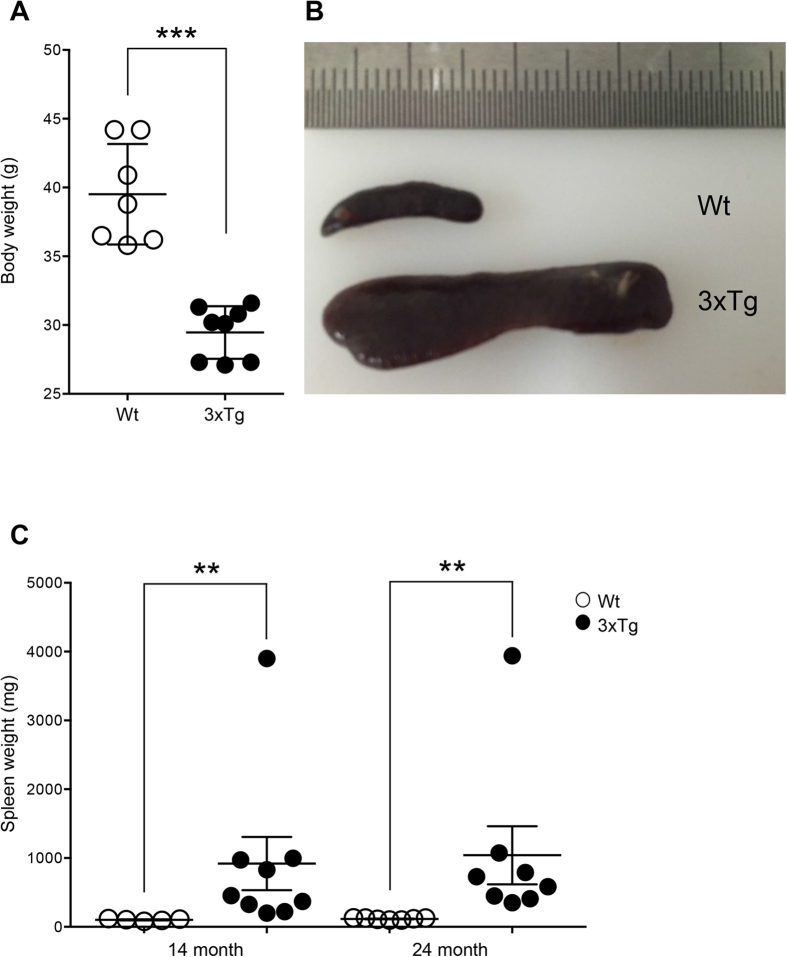
Body weight change and enlargement of the spleen in senile 3xTg-AD mice. (**A**) Body weights of 3xTg-AD mice were compared with wild-type (Wt) mice at the age of 24 months. (**B**) Gross appearance of the spleen in the indicated mice at the age of 24 months. (**C**) Comparison and kinetic analysis of the spleen weight in 3xTg-AD mice as an indicated age. One-way ANOVA followed by Bonferroni’s post-hoc comparisons tests were performed in all statistical analyses (*P < 0.05, **P < 0.01, ***P < 0.001).

We noticed relatively smaller body size and less movement in 3xTg-AD mice compared with wild-type mice at the age of 14 months (data not shown). The 24-month-old 3xTg-AD mice displayed a dramatic decrease in their body weights compared with the wild-type (Fig. 2A and Supplementary Figure S1A). Moreover, we observed that the spleens of transgenic mice were extensively enlarged in both age groups (Fig. 2B,C). The splenomegaly persisted until the age of 24 months regardless of the gender (Supplementary Figure S1B), and then some of these transgenic mice gradually died.

**Figure 3:**
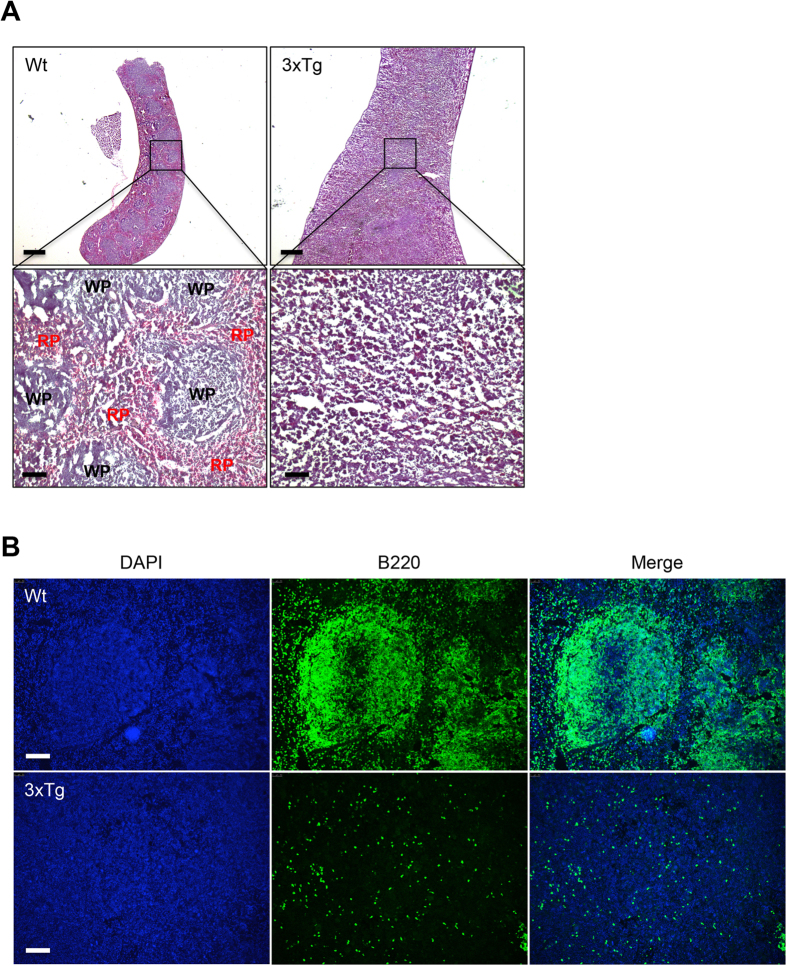
Structural destruction of the spleen in senile 3xTg-AD mice. (**A**) Histological analysis (H&E staining) of the spleen in wild-type (Wt) and 3xTg-AD mice at the age of 24 months. Low magnification; *upper*, High magnification; *lower*. Scale bars = 1 mM *(upper)*, 100 μM (*lower*). (**B**) Immunofluorescence staining with DAPI (which labels the cell nuclei; blue) and B220 (which labels B lymphocytes; green) of the spleen from wild-type (Wt) and 3xTg-AD mice at the age of 24 months. Scale bars = 100 μM.

**Figure 4:**
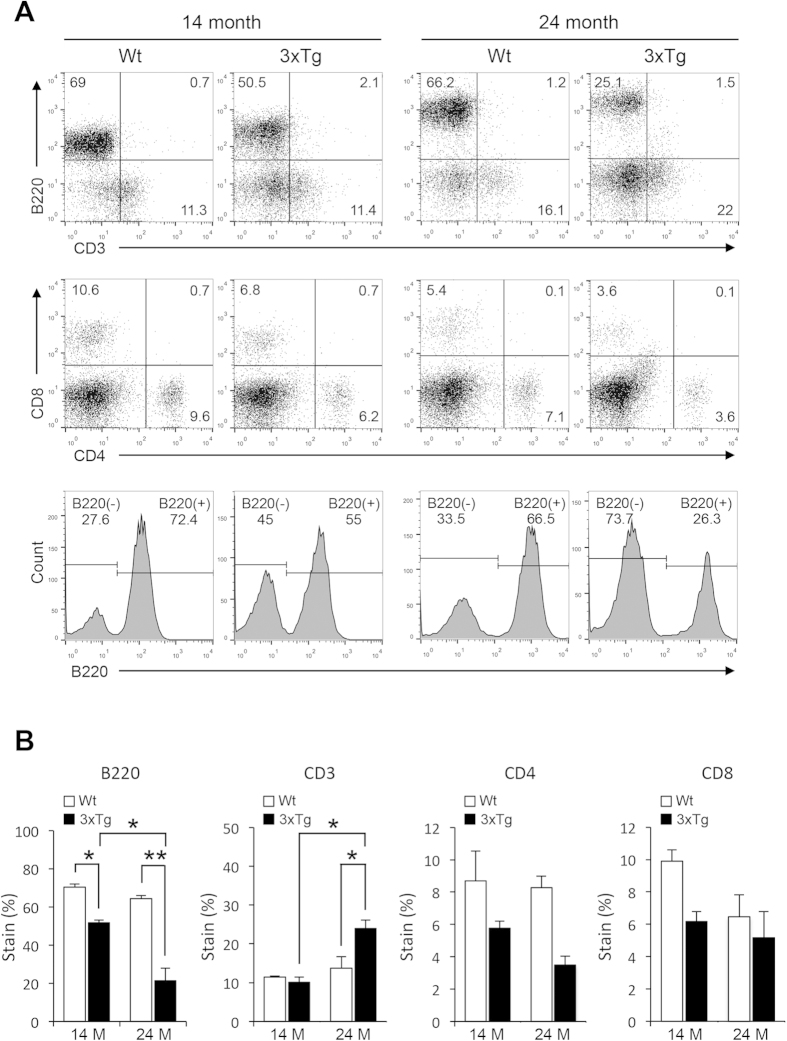
FACS analyses of the spleen in senile 3xTg-AD mice. (**A**) Representative FACS cytogram of CD3 and B220 (*top*), CD4 and CD8 expression pattern (*middle*) and histogram of B220 expression pattern (*bottom*) in splenocytes at the age of 14 and 24 months. (**B**) Relative proportions of T lymphocytes, B lymphocytes and their subpopulations. One-way ANOVA followed by Bonferroni’s post-hoc comparisons tests were performed in all statistical analyses (*P < 0.05, **P < 0.01, ***P < 0.001).

To investigate any compositional change in the enlarged spleen of senile 3xTg-AD mice, we examined the structure of the spleen. Microscopic analyses through Hematoxylene and Eosin (H&E) staining revealed destroyed splenic microarchitecture of senile 3xTg-AD mice characterized by loss of the white pulp and red pulp (Fig. 3A). In immunohistological analyses of B lymphocytes stained with B220 antibody, a normal segregation of B lymphocytes in the white pulp disappeared in the spleen of these mice (Fig. 3B). To further examine the cellular changes in the spleens of 14- and 24-month-old 3xTg-AD mice, FACS analyses were performed. In 14-month-old mice, a statistically significant decrease in the B lymphocytes population (B220(+)) was observed in the spleen while the total T lymphocytes population (CD3(+)) remained unchanged. The decrease in the B lymphocytes population was more dramatic in the spleen of 24-month-old 3xTg-AD mice along with a slight increase in total T lymphocytes population. In contrast to the increase of total T lymphocytes, the helper T lymphocytes (CD4(+)) or cytotoxic T lymphocytes (CD8(+)) were slightly decreased in both 14- and 24-month-old mice although the difference was not statistically significant (Fig. 4A,B). Because APP_swe_ and PS1_M146V_ transgenes are expressed but not translated in the spleen, the possibility of developing splenomegaly due to the functional consequences of the inserted transgenes is unlikely (Supplementary Figure S2). These findings suggest that the structural destruction of the spleen in senile 3xTg-AD mice was accompanied by loss of immune lymphocytes, implying that these mice may have severe splenic dysfunctions.

### Cytokine levels were altered in the plasma of senile 3xTg-AD mice

In aforementioned results, the 3xTg-AD mice showed higher plasma Aβ levels at 14 months when compared with age-matched wild-type, but no significant differences were found at 24 months due to declined Aβ levels in the plasma. For reliable blood-based AD diagnosis, we recognized the need for additional biomarkers that can support Aβ measurements in the blood when Aβ levels are no longer different in the wild-type and AD models. Therefore, we hypothesized that the candidates must show significant alterations in both 14- and 24-month-old when compared with age-matched wild-type to be an effective biomarker.

**Figure 5:**
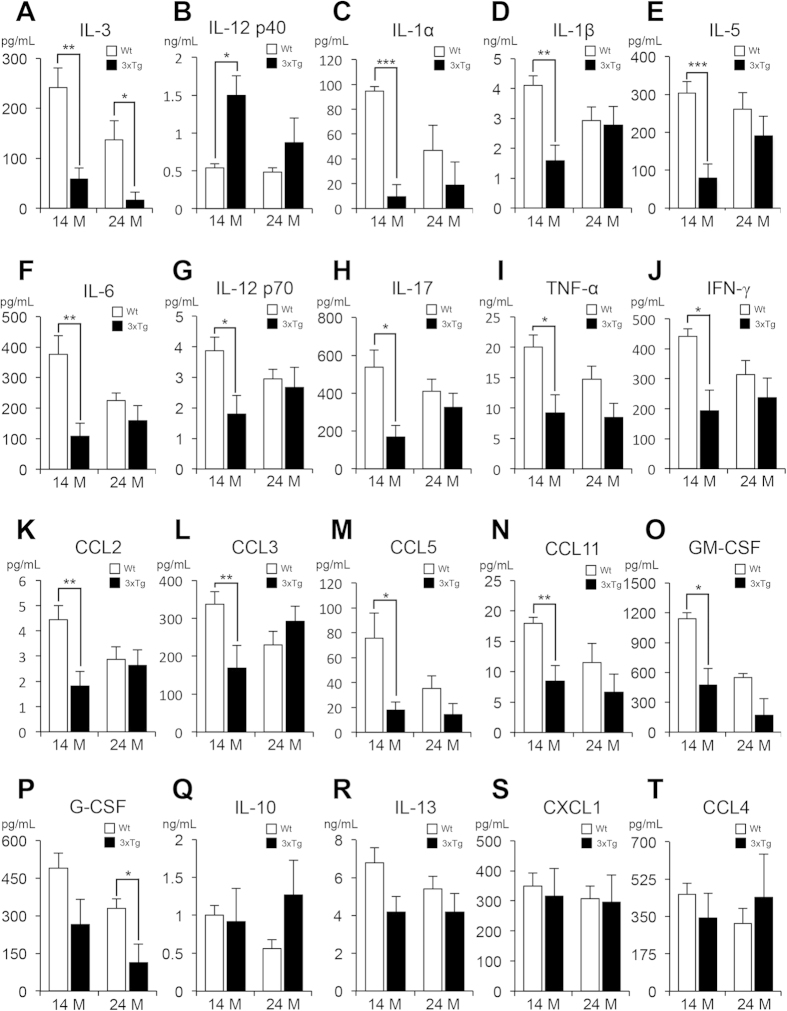
Alterations of cytokine levels in the plasma from wild-type (Wt) and 3xTg-AD mice at the age of 14 months and 24 months. (**A–P**) Significant changes in cytokine levels. (**A**) interleukin-3 (IL-3), (**B**) interleukin-12 p40 (IL-12 p40), (**C**) interleukin-1α (IL-1α), (**D**) interleukin-1β (IL-1β), (**E**) interleukin-5 (IL-5), (**F**) interleukin-6 (IL-6), (**G**) interleukin-12 p70 (IL-12 p70), (**H**) interleukin-17 (IL-17), (**I**) tumor necrosis factor-α (TNF-α), (**J**) interferon-γ (IFN-γ), (**K**) CCL2, (**L**) CCL3, (**M**) CCL5, (**N**) CCL11, (**O**) GM-CSF, (P) G-CSF. (**Q–T**) No difference of cytokine levels between the wild-type and 3xTg-AD mice. (**Q**) interleukin-10 (IL-10), (**R**) interleukin-13 (IL-13), (**S**) CXCL1, (**T**) CCL4. One-way ANOVA followed by Bonferroni’s post-hoc comparisons tests were performed in all statistical analyses (*P < 0.05, **P < 0.01, ***P < 0.001).

Because splenic dysfunction is often associated with altered inflammatory mediator levels, we selected 23 cytokines related to inflammation and measured their concentrations in the plasma of 3xTg-AD mice using the Luminex Bio-Plex cytokine assay kit. The 23 cytokines were interleukin-1α (IL-1α), interleukin-1β (IL-1β), interleukin-2 (IL-2), interleukin-3 (IL-3), interleukin-4 (IL-4), interleukin-5 (IL-5), interleukin-6 (IL-6), interleukin-9 (IL-9), interleukin-10 (IL-10), interleukin-12 p40 (IL-12 p40), interleukin-12 p70 (IL-12 p70), interleukin-13 (IL-13), interleukin-17 (IL-17), tumor necrosis factor-α (TNF-α), interferon-γ (IFN-γ), CXCL1, CCL2, CCL3, CCL4, CCL5, CCL11, G-CSF and GM-CSF. We first compared the concentrations of cytokines in 14- and 24-month-old 3xTg-AD mice to those of respective wild-type mice for evaluation of each cytokine’s diagnostic potential. Of 23 cytokines, only IL-3 displayed statistically significant decline in concentration for both 3xTg-AD age groups when compared with their age-matched wild-type (Fig. 5A). Unlike alterations in Aβ levels, which changed in an age-dependent manner, IL-3 measurements showed the distinction between the wild-type and transgenic models in both age groups.

In addition, the level of IL-12 p40 was increased (Fig. 5B), but those of IL-1α, IL-1β, IL-5, IL-6, IL-12 p70, IL-17, TNF-α, IFN-γ, CCL2, CCL3, CCL5, CCL11 and GM-CSF were all decreased in the 14-month-old transgenic mice (Fig. 5C–O). In 24-month-old 3xTg-AD mice, only G-CSF showed decreased concentration in addition to IL-3 (Fig. 5P). No significant differences were observed in the levels of IL-10, IL-13, CXCL1 and CCL4 in both 3xTg-AD age groups (Fig. 5Q–T). IL-2, IL-4 and IL-9 were excluded from the results because their concentrations were not detectable (data not shown). The 14- and 24-month-old 3xTg-AD mice were then compared to confirm each cytokine’s prognostic potential. Unfortunately, none of the 23 cytokines provided statistically significant results to warrant prognostic potential. Collectively, these results imply that IL-3 may be a promising candidate to support Aβ(1-42) in the blood-based AD diagnosis because it showed altered concentrations in both 14- and 24-month-old groups.

## Discussion

In 14- and 24-month-old 3xTg-AD mice, we found lowered IL-3 levels in the plasma compared with their age-matched wild-type suggesting diagnostic potential of IL-3 for AD diagnosis. We also detected an age-dependent decrease in CSF Aβ(1–42) level, which resembles longitudinal observations in AD patients^32^,^33^,^34^. Similar age-dependent decrease in Aβ(1–42) level was detected in the plasma as well indicating its prognostic potential. Our findings are consistent with previously reported clinical indications that IL-3 is one of AD plasma predictors^35^. Collectively, our results suggest plasma biomarkers such as IL-3 may be able to support the use of plasma Aβ(1–42) in the development of blood-based Alzheimer tests.

Our results regarding changes in splenic T lymphocytes population and cytokine production in 3xTg-AD mice differ from previous studies in a few ways. First, 12-month-old mice from one study exhibited reduced CD3 positive T lymphocytes population while our mice at 14 months showed no significant changes in their total T lymphocytes population^28^. Therefore, how splenic T lymphocytes population change in the 3xTg-AD mice with splenomegaly needs to be further investigated. In addition, report of elevated cytokines levels in the spleen and brain of 3xTg-AD mice warrants future research on whether cytokine levels alter differently in the plasma and in the spleen and brain^36^.

Although the 3xTg-AD mouse is a widely used animal model for the benefit of expressing amyloid plaques, tau tangles and cognitive impairments^25^,^26^,^27^, it is beyond the scope of this study to expect similar splenic abnormalities in AD patients. In addition, the findings from this study may not be applicable to other familial AD mouse models. For example, *ApoE* deficient lupus mouse models are known to have both splenomegaly and B lymphocyte alterations. However, since 3xTg-AD mouse model harbors mutations not only on APP and PS1 but also on tau genes and because splenomegaly or changes in cytokines and lymphocytes have not been yet reported in other AD mouse models such as APP or APP/PS1 transgenic mice, we cannot exclude the implication of tau mutation in splenic dysfunction^37^,^38^.

Amyloid-PET imaging alone is not sufficient for AD diagnosis because of cases including non-demented individuals with amyloid-PET positive^15^,^39^. Thus, additional examination such as neuropsychological tests, brain atrophy observation and hypermetabolism are required for accurate diagnosis of AD. Recently developed tau-PET is considered as one of solutions for aforementioned additional tests^17^,^40^. In the investigation of target candidates for less-invasive and convenient AD diagnosis, the quantification of plasma Aβ level is currently regarded as a promising blood-based biomarker for AD^21^,^41^. However, based on the lesson from amyloid-PET, discovery of additional plasma biomarkers is favorable to enhance the sensitivity and selectivity of AD blood-based test. Because the plasma IL-3 in AD patients has not been studied extensively and the results are contradicting, additional studies are warranted to determine whether our results from animal studies will translate into medical use.

## Methods

### Animals

Triple APP_swe_, PS1_M146V_ and Tau_P301L_ transgenic mice (3xTg-AD) and wild-type (C57Bl/6) mice were obtained from Jackson Laboratory (Bar Harbor, Maine, USA) and then bred in a laboratory animal breeding room at the Korea Institute of Science and Technology. The mice were maintained at constant temperature with an alternating 12 hours light-dark cycle. Food and water were available *ad libitum*. Twenty-nine mice were assessed in this study; 14-months-old 3xTg-AD (n = 9; male 4, female 5) and wild-type (n = 5; male 3, female 2), 24-months-old 3xTg-AD (n = 8; male 4, female 4) and wild-type (n = 7; male 4, female 3). All animal experiments were performed in accordance with the National Institutes of Health guide for the care and use of laboratory animals (NIH Publications No. 8023, revised 1978). The animal studies were approved by the Institutional Animal Care and Use Committee of Korea Institute of Science and Technology.

### Antibodies

The following PE or allophycocyanin conjugated monoclonal antibodies were used for FACS analysis and immunostaining; hamster anti-mouse CD3 (total T lymphocytes), rat anti-mouse CD8 (cytotoxic T lymphocytes) from BD Pharmingen, rat anti-mouse CD4 (helper T lymphocytes) from BioLegend and rat anti-mouse B220 (B lymphocytes) from eBioscience. Alexa-488 conjugated secondary antibody against rat from Invitrogen was applied for fluorescence immunostaining.

### Histology and Immunostaining

Mice were anesthetized with 2% avertin (20 μg/g, i.p.). Removed spleen samples were weighed and fixed in 4% paraformaldehyde (pH 7.4) and immersed in 30% sucrose for cryoprotection. The fixed spleen samples were cut at 10 μm using a Cryostat (Microm HM 525, Thermo Scientific, Waltham, MA, USA) and mounted onto glass slides (Superfrost^**®**^ Plus, Thermo Scientific). The sliced spleens of mice were stained with hematoxylene and eosin (H&E) by H&E staining kit from American MasterTech as following the manufacturer’s instructions. For B lymphocytes staining, the slides were stained by monoclonal antibody (B220). DAPI was used for visualization of nuclei. The images were taken on a Leica DM2500 fluorescence microscope^42^.

### FACS analysis

The alterations of cell populations in the spleen were determined by Immune-fluorescent staining. Spleens were mechanically disrupted by their passage though a syringe and single-cell suspensions were obtained by filtration through cell strainers (75 μm; BD Biosciences). Cells were stained for 15 min at 4 °C with appropriate antibodies in 100 μl of staining buffer (2% FBS, 2 mM EDTA and 0.05% sodium azide in phosphate saline buffer). A FACSort device (BD Bioscience) was used for flow cytometry and FlowJo software (BD Bioscience) for data analysis. Dead cells were gated out by staining with 7-aminoactinomycin D (1 μg/ml) during data collection.

### Luminex bead assay

Under 2% avertin (20 μg/g, i.p.) anesthesia, CSF and blood sampling was performed in accordance with the method described previously^43^. Mice were placed prone, and their cisterna magna were surgically exposed. The exposed meninges were penetrated with laboratory-produced capillary tube that had a tapered tip to obtain CSF. About 5 μL of CSF samples were obtained from each mouse. Then, whole blood samples were collected from the inferior vena cava. Blood plasma and CSF from wild-type and 3xTg-AD mice were assessed for the levels of inflammatory mediators such as cytokines, chemokines and growth factors using a Luminex Bio-Plex cytokine assay kit (Bio-Rad, Richmond, CA) following the manufacturer’s instructions.

### Sandwich-ELISA for detection of Aβ(1–42) in plasma and CSF

Concentration of Aβ(1–42) in the plasma and CSF were measured by using the Human Aβ42 Ultrasensitive ELISA kit (Invitrogen, Cat No. #KHB3544). Detection limit of the kit is less than 1.0 pg/ml. We diluted the collected plasma 30-fold and CSF samples 300-fold. The sandwich-ELISA was performed according to the manufacturer’s instructions using the diluted samples.

### Statistical analysis

Graphs were obtained with GraphPad Prism 6, and the statistical analyses were performed with one-way ANOVA followed by Bonferroni’s post-hoc comparisons (*P < 0.05, **P < 0.01, ***P < 0.001). The error bars represent the SEMs.

## Additional Information

**How to cite this article**: Yang, S.H. *et al*. Abnormalities of plasma cytokines and spleen in senile APP/PS1/Tau transgenic mouse model. *Sci. Rep*. **5**, 15703; doi: 10.1038/srep15703 (2015).

## Supplementary Material

Supplementary Information
