# Supplementary material for: Abnormalities of plasma cytokines and spleen in senile APP/PS1/Tau transgenic mouse model
Source: Sci Rep. 2015 Oct 27;5:15703. doi: 10.1038/srep15703 (PMC4621607; doi:10.1038/srep15703)

SUPPLEMENTARY INFORMATION

Abnormalities of plasma cytokines and spleen in senile APP/PS1/Tau transgenic mouse model

Seung-Hoon Yang, Jiyoon Kim, Michael Jisoo Lee & YoungSoo Kim

Supplementary methods

Genotyping

PCR analysis of genomic DNA from the spleen and westrern blot of total lysate from the brain and spleen were used for genotyping of 3xTg-AD mice as previously described[1,2]. For APP transgene, splenic DNA was isolated by genomic DNA lysis buffer (100 mM Tris, 200 mM NaCl, 5 mM EDTA, 0.2% SDS), and then was amplified by PCR analysis. Expected product of APP transgene is 377 bp. For PS1 transgene, splenic DNA was digested by BstEII restriction enzyme after DNA extraction. Total PCR product of PS1 transgene is 530 bp. After BstEII enzyme digestion, expected product sizes of PS1 transgene are 350 bp and 180 bp. For western blot, the brain and spleen were lysed in tissue lysis buffer (1% SDS, 10 mM Tris). The lysates were separated by gel electrophoresis and transferred onto nitrocellulose membrane. Primary antibody was mouse anti-amyloid-β (6E10 clone, Covance). Blots were developed with the ECL solution (Bio-Rad) following the manufacturer instruction manual.

Supplementary figure legends

Supplementary figure S1. Reduced body weight and increased spleen weight. (A) Gender-matched individual body weight values in the wild-type (Wt) and 3xTg-AD mice at the age of 24 months. (B) Gender-matched individual spleen weight values in the wild-type (Wt) and 3xTg-AD mice at that age of 14 and 24 months.

Supplementary figure S2. Genotypes of 3xTg-AD mice. (A) Expression of APP1 and PS1 transgene in the spleen of wild-type (Wt) and 3xTg-AD mice at the age of 14 months. (B) APP and Tau protein expression levels in the brain and spleen of wild-type (Wt) and 3xTg-AD mice at the age of 14 months.

Supplementary references

1 Guo, Q. *et al.* Increased vulnerability of hippocampal neurons to excitotoxic necrosis in presenilin-1 mutant knock-in mice. *Nat Med* **5**, 101-106, doi:10.1038/4789 (1999).

2 Oddo, S. *et al.* Triple-transgenic model of Alzheimer's disease with plaques and tangles: intracellular Abeta and synaptic dysfunction. *Neuron* **39**, 409-421 (2003).

Supplementary Figure S1

A

24 month			
Wt (g)		3xTg (g)	
Gender	Weight (g)	Gender	Weight (g)
male	40.9	male	27.3
male	44.2	male	31.3
male	44.2	male	30.8
male	38.8	male	30.2
female	36.2	female	27.3
female	35.8	female	30.1
female	36.5	female	31.6
		female	27.1

B

14 month				24 month			
Wt		3xTg		Wt		3xTg	
Gender	Weight (mg)	Gender	Weight (mg)	Gender	Weight (mg)	Gender	Weight (mg)
male	112	male	455	male	129	male	791
male	81	male	831	male	128	male	730
male	93	male	997	male	132	male	448
female	121	male	326	male	98	male	583
female	104	female	3900	female	98	female	350
		female	973	female	109	female	3940
		female	368	female	117	female	408
		female	200			female	1072
		female	222				

Supplementary Figure S2

A

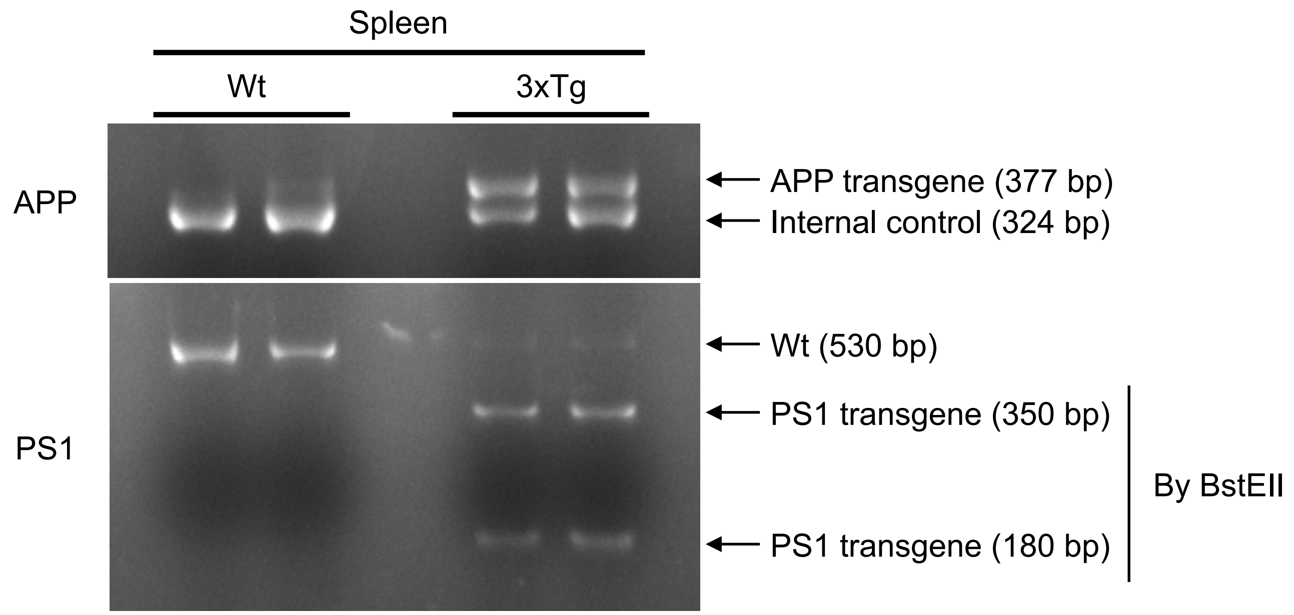

B

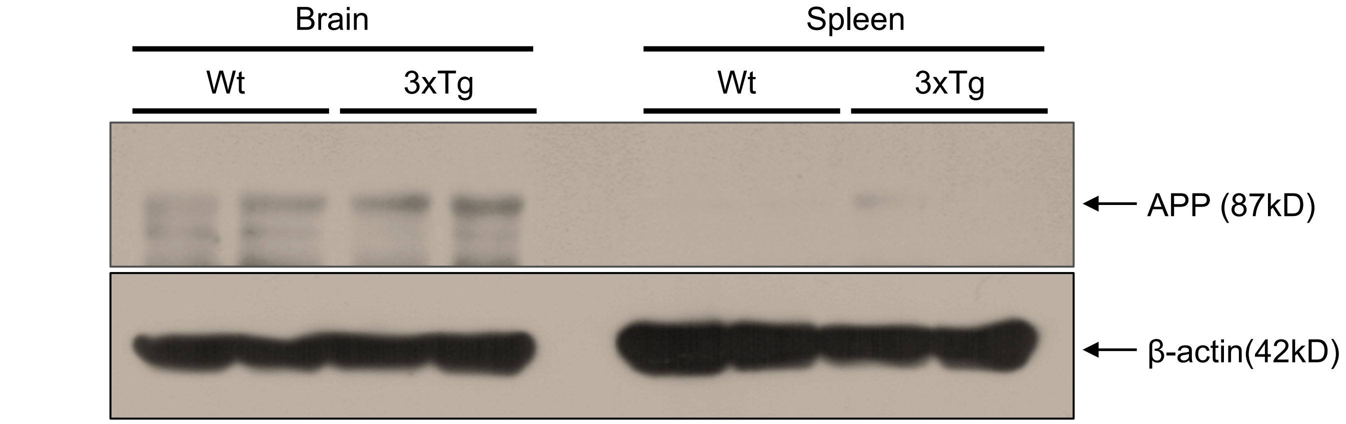

Supplement: Supplementary Information [file srep15703-s1.pdf]
